# Changes in frequency of park/playground utilization among children aged 4–59 months in Los Angeles County, California 2008–2020

**DOI:** 10.1016/j.pmedr.2022.101976

**Published:** 2022-09-06

**Authors:** Christopher E. Anderson, Shannon E. Whaley

**Affiliations:** Division of Research and Evaluation, Public Health Foundation Enterprises WIC, a Program of Heluna Health, 12781 Schabarum Ave, Irwindale, CA 91706, USA

**Keywords:** Park, Playground, Physical activity, COVID-19, Disparities, Play, Child health, CI, confidence interval, FPL, federal poverty level, LAC, Los Angeles County, OR, odds ratio, USD, United States dollars, WIC, the Special Supplemental Nutrition Program for Women, Infants, and Children

## Abstract

Child physical activity and play are critical for healthy development, and parks/playgrounds are important public spaces that provide physical activity/play opportunities. This study was conducted to assess changes in park/playground utilization by Special Supplemental Nutrition Program for Women, Infants and Children (WIC)-participating children from 2008 to 2020, and whether the COVID-19 pandemic was associated with lower park/playground utilization and racial/ethnic disparities in park/playground utilization. Cross-sectional data from the 2008–2020 triennial Los Angeles County WIC Survey (n = 21,886) were used, and analyses stratified by child age (4–23 months, 24–59 months). Odds ratios (OR) and 95 % confidence intervals (CI) for the relationship between year and park/playground utilization frequency were determined from multinomial logistic regression, and racial/ethnic disparities were assessed by interacting year with race/ethnicity. Among children 24–59 months of age, park/playground utilization increased compared to never from 2011 to 2017 compared to 2008 (Every day, 2011–2017: OR [95 % CI]: 2.69 [1.93, 3.75], 4.71 [3.23, 6.86], 10.20 [6.91, 15.06]; 3–6 days/week 2011–2017: 1.54 [1.13, 2.10], 3.11 [2.18, 4.45], 3.94 [2.71, 5.72]; 1–2 days/week, 2014–2017; 1.53 [1.08, 2.18], 1.63 [1.13, 2.37]). Associations reversed in 2020, with 36 % lower odds of every day (OR [95 % CI]: 0.64 [0.48, 0.85]), 85 % lower odds of 3–6 days/week (0.15 [0.11, 0.20]) and 89 % lower odds of 1–2 days/week (0.11 [0.09, 0.15]) park/playground utilization compared to never than in 2008. Park/playground utilization frequency increased from 2008 to 2017, but progress reversed during the COVID-19 pandemic in 2020. Results for children ages 4–23 months were similar. Future public health restrictions to public recreation facilities should consider realistic limitations to potential benefits and the potential for unintended consequences before implementation.

## Highlights

• Every day and 3–6 d/wk park use rose from 2008 to 2017, and declined in 2020.
• Never park use declined from 2008 (12.2%) to 2017 (8.5%) and rose in 2020 (35.5%).
• Children 24–59 mo had 36% lower every day park use odds in 2020 than 2008.
• Children 24–59 mo had 85–89% lower 3–6 and 1-2d/wk park use odds in 2020 than 2008.
• Children 4–23 mo had similar patterns in park utilization frequency 2008–2020.

## 1 Introduction

Physical activity is critical for the promotion and maintenance of good health (Bauman, 2004). Childhood physical activity, including play, contributes to healthy psychological, social and emotional development (American Academy of Pediatrics Council on Sports Medicine and Fitness and Council on School Health, 2006, Ginsburg, 2007, Pellegrini and Smith, 1998), and to healthy activity patterns through childhood to adulthood (Janssen and Leblanc, 2010). The majority of children in the United States fall short of the 60 min of daily moderate to vigorous physical activity that is recommended (National Physical Activity Plan, 2016), and activity rates are low among children irrespective of gender, race, age and socioeconomic status (Whitt-Glover et al., 2009).

The physical environment influences physical activity (Owen et al., 2000, Spence and Lee, 2003), and parks/playgrounds are public spaces that provide opportunities for children to interact, engage in activity and play (Bedimo-Rung et al., Feb 2005, Kaczynski and Henderson, 2007). Children in low-income households and communities have more restricted access to high-quality parks and playgrounds in their neighborhoods than children in higher-income households and communities (McKenzie et al., 2013, Razani et al., 2020), contributing to fewer physical activity opportunities (Tandon et al., 2012) and higher rates of obesity (Nobari et al., 2018) among children from low-income households.

Children participating in the Special Supplemental Nutrition Program for Women, Infants, and Children (WIC) reside in households with incomes below 185 % of the Federal Poverty Level (FPL) (Oliveira and Frazao, 2015), and 14 % of WIC participating children ages 2–4 years were obese in 2016 (Pan et al., 2019). The COVID-19 pandemic, and associated governmental restrictions on access to public spaces during 2020, have contributed to concerns about potential physical activity declines and consequences for obesity and general health among children (Slater et al., 2020). Physical activity declines were identified in a review of 21 studies on youth physical activity during the COVID-19 pandemic (Yomoda and Kurita, 2021). In Los Angeles County (LAC), California, playgrounds and other facilities in public parks were ordered closed on March 23, 2020 (Los Angeles County Department of Parks and Recreation, 2020), with many playgrounds roped off, and playgrounds were subject to stringent reopening requirements through November 23, 2020 including social-distancing from all individuals from outside the household, mask wearing for children 2 years and older, and time limits on visits (Los Angeles County Department of Public Health, 2020). Given COVID-19-related restrictions, limited household resources and high obesity prevalence, understanding patterns of park/playground utilization as a proxy for physical activity and access to play among WIC participants is critical. This study was conducted to evaluate the frequency of park/playground utilization from 2008 to 2020 among LAC WIC participants, and to determine if racial/ethnic disparities in frequency of park/playground utilization were present before or during the COVID-19 pandemic. We hypothesized that frequency of park/playground utilization would be lower in 2020 relative to prior years, and that racial/ethnic disparities in frequency of park/playground utilization would increase in 2020 relative to prior years.

## 2 Methods

### 2.1 Setting and subjects

WIC is a federal nutrition assistance program that provides supplemental food packages, nutrition education, breastfeeding support and health and social service referrals to pregnant and postpartum women, and infants and children to 5 years of age, who live in low-income households (Oliveira and Frazao, 2015, United States Department of Agriculture, 2020). LAC is home to the largest local WIC-participant population in the nation. This study includes data from 21,886 WIC participants 4–59 months of age and their mothers who responded during administrations of the triennial LAC WIC Survey in 2008 (n = 2,668), 2011 (n = 4,984), 2014 (n = 4,295), 2017 (n = 4,638), and 2020 (n = 5,301) (Data Mining Project, 2020). Respondents in each of the five unique cross-sectional samples received incentives of 10 United States dollars (USD) for completing the computer assisted telephone survey, and response rates were 60, 54, 50, 52 and 53 % for 2008, 2011, 2014, 2017 and 2020 administrations, respectively. The California Department of Health and Human Services Institutional Review Board approved this study, and oral informed consent was obtained from all study participants.

### 2.2 Data sources

Mothers responding to the LAC WIC Survey answer questions about pregnancy and birthing experiences, health insurance, healthcare, social service and childcare utilization, social support, household composition, food security and detailed demographics. Survey data are complemented by administrative data, collected during WIC eligibility certification, on participating children (e.g. race/ethnicity, sex, age, nutritional risks), their mothers (e.g. education, language preference), and their households (e.g. address of residence, size, income).

### 2.3 Park use frequency

In all survey administrations, mothers reported the frequency with which the child for whom the survey was completed was taken to a park, playground or other safe place to play (every day, 3–6 days/week, 1–2 days/week, never). Park/playground utilization frequency was validated with 2008 LAC WIC Survey data in which respondents also reported the activity of their child relative to the child’s peers, with the odds of being reported to be more active than one’s peers being 60 % (OR 1.60, 95 % CI 1.25, 2.06), 28 % (OR 1.28, 95 % CI 1.04, 1.59) and 19 % (OR 1.19, 95 % CI 0.98, 1.43) higher among children reported to visit a park/playground every day, 3–6 days/week and 1–2 days/week, respectively, compared to those who never visited a park/playground. Responding mothers also reported their perception of the safety of their neighborhood of residence (excellent, good, fair, poor). In the 2008 survey administration, community safety was assessed specifically with regard to neighborhood safety *from crime* (response options: very safe, somewhat safe, somewhat unsafe, not at all safe). Questions for park use and perceived safety were based upon items from the 2007 Los Angeles County Health Survey (Los Angles County Department of Public Health, Los Angeles County Health Survey, 2007).

### 2.4 Other variables

The primary exposure of interest in this analysis is the year of survey administration (2008, 2011, 2014, 2017 and 2020). Covariates evaluated for inclusion in the analysis included child sex, age (4–11, 12–23, 24–35, 36–47, 48–59 months), and race/ethnicity (Asian, Black, English-speaking Hispanic, Spanish-speaking Hispanic, White, Other); maternal age, body mass index (weight (kg)/ height (m)^2^) from self-reported weight and height, educational attainment (<high school degree, high school degree, >high school degree), employment (working for pay: yes, no) and childcare use (yes, no); household income (<1,200, 1,200-<1,800, 1,800–2,400 and greater than 2,400 USD per month); paternal presence (yes, no) and employment (working for pay: yes, no). Address of residence from WIC administrative data was geocoded, and the census tract of residence was determined for each survey respondent. Population density (residents/ area (mile^2^)) and the percent of residents in each census tract living below the federal poverty level were determined based upon 5-year American Community Survey data for the 5-year period ending closest to the year of the survey administration (US Census Bureau, 2021).

### 2.5 Statistical analysis

Study participant characteristics were summarized and statistical comparisons were made between survey years using frequencies, proportions and chi-square tests for categorical variables and means, standard deviations and analysis of variance F tests for continuous variables. Covariates for regression models were selected *a priori* based upon child, maternal, household and neighborhood characteristics with previously reported associations with child PA. The association between survey year and park/playground utilization frequency was assessed with generalized estimating equation multinomial logistic regression models, stratified by child age (4–23 months, 24–59 months), accommodating multiple surveys within families (n = 305 families with repeated surveys) and clustering within census tracts (n = 1,967 census tracts). Odds ratios (OR) and corresponding 95 % confidence intervals (CI) represent the odds of being in a specified category of park/playground utilization frequency (every day, 3–6 days/week, 1–2 days/week) compared to the never category in the specified survey year relative to 2008. This model included independent terms for child age, sex and race/ethnicity; maternal age, educational attainment, employment status and neighborhood safety perception; census tract population density and percent of residents living below the federal poverty level; and survey year. Racial/ethnic disparities were evaluated before and during the COVID-19 pandemic (dichotomous: 2008–2017, 2020), in a similarly parameterized model with the addition of a two-way interaction of child race/ethnicity and survey year (dichotomous).

Two sensitivity analyses were undertaken. The first evaluated robustness of the observed associations to community safety perception by removing the independent variable for community safety perception from regression models. The second determined whether seasonality (time of year the surveys were collected) influenced observed associations by comparing the proportion of respondents in each category of park/playground utilization frequency by month of survey collection in the 2020 sample, controlling for child age and race/ethnicity. All analyses were conducted using SAS 9.4 (SAS Institute Inc., Cary, NC, USA). P-values<0.05 were considered statistically significant.

## 3 Results

Characteristics of WIC-participating children included in this study are presented in Table 1. Around half of survey subjects were male, and a majority were Hispanic in every year of the survey administration. The frequency of park/playground visitation increased from 2008 to 2017 and decreased in 2020 (p-value < 0.001), while the perception of community safety was higher in 2008 than all subsequent years (p-value < 0.001). The proportion of mothers who were ≥ 35 years of age, had a BMI of ≥ 30 kg/m^2^, and had education > high school degree increased significantly from the first to the most recent survey, reaching 32.1 %, 43.0 % and 46.0 %, respectively, in 2020 (p-values < 0.001). The majority of mothers were not working for pay and the majority of households included the child’s father in all survey years. The proportion of households reporting incomes < 1,800 USD/month was highest in 2011 and 2014 surveys, with over 70 % of households reporting income < 1,800 USD/month in those years (p-value < 0.001). The proportion of residents living in poverty in survey respondents’ neighborhoods increased from 29.2 % in 2008 to 35.3 % in 2020 (p-value < 0.001) while the neighborhood population density increased from 16.0 thousand people/square mile in 2008 to 17.2 thousand people/square mile in 2020 (p-value < 0.001).

**Table 1:** Characteristics of WIC-participating children, their mothers and households from the 2008–2020 Los Angeles County WIC Survey (n = 21,886).

		**2008**	**2011**	**2014**	**2017**	**2020**	
		N = 2,668	N = 4,984	N = 4,295	N = 4,638	N = 5,301	p
**Child traits, n (%)**							
Male		1332 (49.9)	2543 (51.0)	2240 (52.2)	2370 (51.1)	2674 (50.4)	0.38
Race/ethnicity							<0.001
	Asian	62 (2.3)	198 (4.0)	31 (0.7)	94 (2.0)	646 (12.2)	
	Black	164 (6.1)	435 (8.7)	430 (10.0)	326 (7.0)	667 (12.6)	
	Hispanic-SP	818 (30.7)	1951 (39.1)	2003 (46.6)	2377 (51.3)	1961 (37.0)	
	Hispanic-EN	1369 (51.3)	2122 (42.6)	1149 (26.8)	1685 (36.3)	1680 (31.7)	
	White	58 (2.2)	218 (4.4)	614 (14.3)	131 (2.8)	320 (6.0)	
	Other	197 (7.4)	60 (1.2)	68 (1.6)	25 (0.5)	27 (0.5)	
Age, mo							<0.001
	<12	587 (22.0)	1367 (27.4)	883 (20.6)	404 (9.2)	578 (11.8)	
	12 to < 24	589 (22.1)	1020 (20.5)	931 (21.7)	1055 (23.9)	1213 (24.7)	
	24 to < 36	530 (19.9)	918 (18.4)	819 (19.1)	1006 (22.8)	1051 (21.4)	
	36 to < 48	528 (19.8)	920 (18.5)	839 (19.5)	986 (22.3)	1178 (24.0)	
	48 to < 60	434 (16.3)	759 (15.2)	822 (19.1)	961 (21.8)	885 (18.0)	
Park frequency, d/wk							<0.001
	Every day	292 (10.9)	1040 (20.9)	985 (22.9)	1666 (35.9)	1567 (29.6)	
	3 to 6	692 (25.9)	1424 (28.6)	1376 (32.0)	1362 (29.4)	748 (14.1)	
	1 or 2	1358 (50.9)	1843 (37.0)	1419 (33.0)	1214 (26.2)	1106 (20.9)	
	Never	326 (12.2)	677 (13.6)	515 (12.0)	396 (8.5)	1880 (35.5)	
Community safety a							<0.001
	Excellent	930 (34.9)	1074 (21.5)	922 (21.5)	979 (21.1)	1239 (23.4)	
	Good	1313 (49.2)	2274 (45.6)	2044 (47.6)	2220 (47.9)	2541 (47.9)	
	Fair	338 (12.7)	1398 (28.0)	1145 (26.7)	1194 (25.7)	1234 (23.3)	
	Poor	87 (3.3)	238 (4.8)	184 (4.3)	245 (5.3)	287 (5.4)	
**Maternal traits, n (%)**							
Age, yr							<0.001
	17 to 24	765 (28.9)	1507 (30.4)	1120 (26.2)	931 (20.2)	836 (15.9)	
	25 to 34	1264 (47.8)	2426 (49.0)	2132 (49.9)	2425 (52.6)	2742 (52.0)	
	35 to 60	614 (23.2)	1023 (20.6)	1021 (23.9)	1256 (27.2)	1693 (32.1)	
BMI, kg/m^2^							<0.001
	<25	969 (36.4)	1742 (36.2)	1389 (32.4)	1371 (29.7)	1399 (26.6)	
	25 to < 30	954 (35.8)	1584 (32.9)	1410 (32.9)	1551 (33.5)	1600 (30.4)	
	≥30	742 (27.8)	1482 (30.8)	1490 (34.7)	1701 (36.8)	2258 (43.0)	
Education							<0.001
	< HS degree	1210 (45.4)	1740 (34.9)	1363 (31.7)	1486 (32.0)	1282 (24.2)	
	HS degree	724 (27.1)	1518 (30.5)	1263 (29.4)	1320 (28.5)	1578 (29.8)	
	>HS degree	734 (27.5)	1726 (34.6)	1669 (38.9)	1832 (39.5)	2441 (46.0)	
Not working for pay		1766 (66.2)	3352 (67.3)	2634 (61.3)	2624 (56.6)	3275 (61.8)	<0.001
Childcare use		963 (36.1)	1835 (36.8)	1746 (40.7)	1879 (40.5)	1704 (32.1)	<0.001
**Household traits, n (%)**							
Income, dollars/mo							<0.001
	<1,200	934 (35.0)	2037 (40.9)	1746 (40.7)	1491 (32.1)	1630 (30.7)	
	1,200 to < 1,800	871 (32.6)	1570 (31.5)	1331 (31.0)	1356 (29.2)	1660 (31.3)	
	1,800 to < 2,400	451 (16.9)	749 (15.0)	638 (14.9)	887 (19.1)	1285 (24.2)	
	≥2,400	412 (15.4)	628 (12.6)	580 (13.5)	904 (19.5)	726 (13.7)	
Father present		1795 (67.3)	3295 (66.1)	2719 (63.3)	2942 (63.4)	3356 (63.3)	<0.001
Father not working for pay		191 (10.6)	623 (18.9)	396 (14.6)	345 (11.7)	708 (21.1)	<0.001
**Neighborhood**^b^**population, mean ± SD**							
Poverty, % of residents		29.2 ± 11.6	29.2 ± 12.0	30.6 ± 11.8	37.8 ± 12.5	35.3 ± 12.5	<0.001
Density 1,000 s/ sq mi		16.0 ± 11.0	15.7 ± 10.5	16.2 ± 10.6	17.1 ± 11.6	17.2 ± 11.9	<0.001

Abbreviations: d = days; EN = English-speaking; HS = high school; mi = mile; mo = months; SP = Spanish-speaking; sq = square; WIC = the Special Supplemental Nutrition Program for Women, Infants and Children; wk = week; yr = years;

a Community safety was reported by the child’s mother. In the 2008 survey, response options related specifically to general neighborhood perceived safety *from crime* (response options: very safe, somewhat safe, somewhat unsafe, not at all safe), while 2011–2020 surveys assessed general neighborhood perceived safety (response options: excellent, good, only fair, poor).

b Neighborhood was assessed as the census tract of residence. Data for neighborhood characteristics were drawn from the 5-year estimates from the American Community Survey for the year of the survey.

ORs for frequency of park utilization by year of survey administration and by child age are presented in Table 2. Similar patterns were observed in both child age strata so only ages 24–59 months are discussed here. ORs indicate substantial increases in every day and 3–6 days/week categories relative to never from 2008 to 2011, 2014 and 2017, and substantial increases for the 1–2 days/week category relative to never from 2008 to 2014 and 2017, followed by a significant reversal of the trend and decreases for all categories relative to never in 2020. The odds of being in the every day category relative to the never category were 169 % higher in 2011 (OR [95 % CI]: 2.69 [1.93, 3.75]), 371 % higher in 2014 (4.71 [3.23, 6.86]), 920 % higher in 2017 (10.20 [6.91, 15.06]), and 36 % lower in 2020 (0.64 [0.48, 0.85]) than in 2008. The odds being in the 3–6 day/week category relative to the never category were 54 % higher in 2011 (1.54 [1.13, 2.10]), 211 % higher in 2014 (3.11 [2.18, 4.45]), 294 % higher in 2017 (3.94 [2.71, 5.72]), and 85 % lower in 2020 (0.15 [0.11, 0.20]) than in 2008. The odds of being in the 1–2 days/week category relative to never were 53 % higher in 2014 (1.53 [1.08, 2.18]), 63 % higher in 2017 (1.63 [1.13, 2.37]), and 89 % lower in 2020 (0.11 [0.09, 0.15]) than in 2008.

**Table 2:** Odds ratios a for frequency of park/playground utilization by survey year, relative to 2008.

Frequency	2008^a^	2011 a	2014 a	2017 a	2020 a
	Ages 24 to 59 months				
7 d/wk	1.00 (ref)	**2.69 (1.93, 3.75)**	**4.71 (3.23, 6.86)**	**10.20 (6.91, 15.06)**	**0.64 (0.48, 0.85)**
3–6 d/wk	1.00 (ref)	**1.54 (1.13, 2.10)**	**3.11 (2.18, 4.45)**	**3.94 (2.71, 5.72)**	**0.15 (0.11, 0.20)**
1–2 d/wk	1.00 (ref)	0.91 (0.67, 1.23)	**1.53 (1.08, 2.18)**	**1.63 (1.13, 2.37)**	**0.11 (0.09, 0.15)**
	Ages 4 to 23 months				
7 d/wk	1.00 (ref)	**2.06 (1.55, 2.73)**	**1.80 (1.35, 2.41)**	**4.05 (2.96, 5.55)**	1.04 (0.78, 1.40)
3–6 d/wk	1.00 (ref)	1.11 (0.88, 1.40)	0.88 (0.69, 1.13)	**1.45 (1.10, 1.91)**	**0.16 (0.12, 0.21)**
1–2 d/wk	1.00 (ref)	**0.80 (0.65, 0.97)**	**0.64 (0.52, 0.78)**	0.83 (0.65, 1.07)	**0.14 (0.11, 0.17)**

a OR (95 % CI) reflect the relative odds of being in the specified frequency category relative to the never category in the specified survey year relative to 2008. For example, the relative odds of visiting a park every day compared to never were 2.06 times higher for WIC participants 4 to 23 months of age in 2011 compared to 2008. ORs are from generalized estimating equation multinomial logistic regression models, accommodating clustering of repeated observations within families (n = 305 families with repeated observations) and within census tract of residence and adjusted for child age, sex and race/ethnicity; maternal age, educational attainment, employment status and perception of neighborhood safety; survey year; and census tract population density and percent of residents living below the federal poverty level. Model was stratified by child age, performed separately for children 4 to 23 months of age and 24 to 59 months of age.

**Fig. 1:**
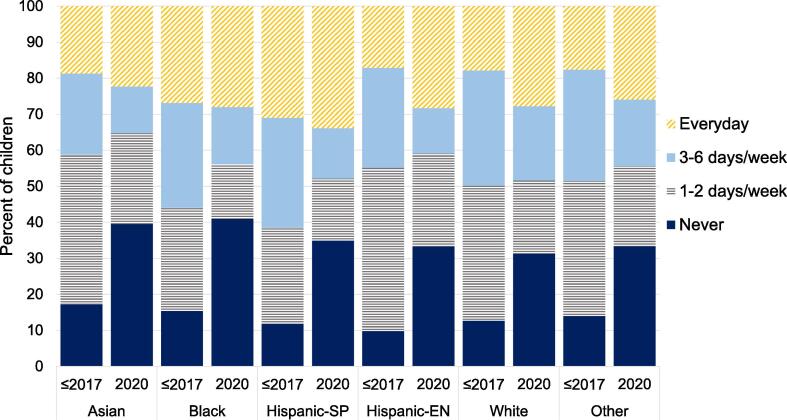
Frequency of park/playground utilization by race/ethnicity prior to 2020 and in 2020.

For all race/ethnicity groups, the proportion of never park/playground users increased in 2020 and the proportion of 1–2 and 3–6 days/week park/playground utilization decreased in 2020 (Fig. 1). Interestingly, the proportion of every day park/playground utilization increased from before to during 2020 in all race/ethnicity groups, and these increases were largest among children from English-speaking Hispanic, White and Other race/ethnicity groups. Spanish-speaking Hispanic children had the highest prevalence of every day park/playground use before and during 2020. Among children 24–59 months of age, Asian children had significantly lower odds of every day, 3–6 days/week and 1–2 days/week park/playground frequency before 2020, and significantly lower odds of every day and 3–6 days/week frequency in 2020 compared to English-speaking Hispanic children (Table 3). Black children demonstrated significantly higher odds of every day park/playground use before 2020, and significantly lower odds of every day and 1–2 days/week park/playground use in 2020 relative to English-speaking Hispanic children. Spanish-speaking Hispanic children demonstrated higher odds of every day park/playground use and lower odds of 1–2 day/week park/playground use compared to English-speaking Hispanic children before 2020, and no differences were observed in 2020. White children demonstrated higher odds of 3–6 days/week park/playground use than English-speaking Hispanic children in 2020, with no differences observed before 2020. Racial/ethnic disparities (relative to English-speaking Hispanic children) in park/playground use frequency among children ages 4–23 months of age in 2020 were either of similar or smaller magnitude compared to before 2020.

**Table 3:** Odds ratios a for the association between race/ethnicity and more frequent park/playground utilization before and during COVID pandemic.

		7 days/week		3–6 days/week		1–2 days/wk	
Race-ethnicity		2008-2017^a^	2020^a^	2008-2017^a^	2020^a^	2008-2017^a^	2020^a^
		Ages 24 to 59 months					
	Asian	**0.31 (0.17, 0.55)**	**0.43 (0.31, 0.60)**	**0.24 (0.14, 0.42)**	**0.61 (0.40, 0.93)**	**0.37 (0.21, 0.63)**	0.86 (0.61, 1.21)
	Black	**1.86 (1.12, 3.06)**	**0.65 (0.47, 0.90)**	1.16 (0.71, 1.89)	0.90 (0.61, 1.32)	0.83 (0.51, 1.36)	**0.55 (0.39, 0.79)**
	Hispanic-SP	**1.90 (1.41, 2.55)**	1.06 (0.83, 1.34)	1.21 (0.91, 1.63)	1.08 (0.80, 1.45)	**0.69 (0.51, 0.92)**	0.79 (0.61, 1.02)
	White	0.93 (0.57, 1.52)	0.91 (0.58, 1.44)	1.07 (0.66, 1.74)	**1.84 (1.15, 2.95)**	0.86 (0.53, 1.38)	0.96 (0.59, 1.56)
	Other	0.52 (0.23, 1.18)	1.37 (0.10, 19.33)	0.54 (0.25, 1.17)	5.89 (0.56, 61.58)	0.55 (0.25, 1.22)	3.72 (0.40, 35.00)
	Hispanic-EN	1.00 (ref)	1.00 (ref)	1.00 (ref)	1.00 (ref)	1.00 (ref)	1.00 (ref)
		Ages 4 to 23 months					
	Asian	0.68 (0.38, 1.20)	**0.55 (0.35, 0.87)**	**0.43 (0.25, 0.73)**	**0.56 (0.31, 0.99)**	**0.52 (0.35, 0.77)**	**0.59 (0.37, 0.92)**
	Black	0.86 (0.64, 1.16)	0.76 (0.49, 1.18)	**0.58 (0.45, 0.77)**	0.82 (0.48, 1.40)	**0.40 (0.31, 0.52)**	**0.40 (0.24, 0.65)**
	Hispanic-SP	**1.55 (1.26, 1.92)**	0.93 (0.65, 1.31)	**0.77 (0.63, 0.93)**	**0.63 (0.40, 0.98)**	**0.54 (0.45, 0.64)**	**0.50 (0.34, 0.72)**
	White	0.77 (0.53, 1.12)	0.99 (0.53, 1.87)	0.72 (0.51, 1.01)	0.82 (0.38, 1.76)	**0.58 (0.44, 0.77)**	0.75 (0.40, 1.40)
	Other	0.93 (0.54, 1.58)	1.71 (0.39, 7.56)	0.90 (0.57, 1.45)	0.75 (0.06, 8.77)	0.66 (0.42, 1.02)	0.66 (0.13, 3.38)
	Hispanic-EN	1.00 (ref)	1.00 (ref)	1.00 (ref)	1.00 (ref)	1.00 (ref)	1.00 (ref)

a OR (95 % CI) reflect the relative odds of being in a specified frequency category relative to the never frequency categories in the specified years for children of specified races compared to English-speaking Hispanic children. ORs are from generalized estimating equation multinomial logistic regression models, accommodating clustering of repeated observations within families (n = 305 families with repeated observations) and within census tract of residence, and are adjusted for child age, sex and race/ethnicity; maternal age, educational attainment, employment status and perception of neighborhood safety; survey year (dichotomous 2020 vs all others); census tract population density and percent of residents living below the federal poverty level; and the interaction of child race and survey year (dichotomous). Model was stratified by child age, performed separately for children 4 to 23 months of age and 24 to 59 months of age.

In the first sensitivity analysis, the magnitude and significance of all associations were robust to the removal of community safety perception from the regression models (data not shown), thus community safety perception was retained in the final models. In the second sensitivity analysis, the odds of children being in each category of park/playground utilization frequency did not differ between months of survey administration (p-value 0.13), suggesting seasonality is not of concern in the repeated cross-sectional analysis.

## 4 Discussion

The frequency of park/playground utilization among WIC-participating children in LAC increased from 2008 to 2011, 2011 to 2014, and 2014 to 2017. This pattern reversed in 2020 following the closure, and in many cases roping off with caution tape, of playgrounds in LAC parks in March 2020 and the imposition of strict reopening protocols for nearly the duration of 2020 (Los Angeles County Department of Public Health, 2020, Los Angeles County Department of Parks and Recreation, 2020). In 2020 fewer children were reported to be in 3–6 days/week or 1–2 days/week categories than in any prior year, more children were reported to be in the never category than in any prior year, and fewer children were reported to be in the every day category than in 2017. The same general pattern of park/playground utilization frequency was apparent among children 4–23 months of age and 24–59 months of age, and among children from all racial/ethnic groups. The declines in utilization frequency were more pronounced among Asian, Black and Spanish-speaking Hispanic children than among English-speaking Hispanic, White and Other race/ethnicity children. The abrupt departure from the long-term trend toward more frequent utilization of parks/playgrounds may have important health implications for low-income children in LAC in the future.

The increase in park/playground utilization from 2008 to 2017 occurred during a period in which the LAC Department of Public Health, the LAC Department of Parks and Recreation, and the Los Angeles Collaborative for Healthy Active Children were managing a physical activity promotion campaign for children called “Get Active, Get Healthy LA!”, involving coordinated efforts since 2010 to promote physical activity among young children in LAC (Los Angeles County Department of Public Health, 2010). Simultaneous efforts to promote physical activity in childcare settings since 2013 were supported by other community organizations (Child Care Alliance Los Angeles, 2022). In an analysis of a nationally-representative sample, over half (58 %) of preschool-aged children played outdoors at least once a day (Tandon et al., 2012). That value exceeds the highest level of every day park/playground utilization reported in the present study (in 2017) of 36 %. The lower frequency reported among WIC-participating children relative to the nationally-representative sample may be due to a slightly different study outcome (WIC: frequency of visiting a park/playground vs National: frequency of any outdoor play) or sociodemographic differences between the samples (higher Hispanic ethnicity prevalence, more mothers not working and fewer mothers with greater than a high school education in WIC sample), given previously reported associations between lower household and neighborhood socioeconomic status and more limited access to public spaces for play such as parks/playgrounds (McKenzie et al., 2013, Razani et al., 2020).

The marked decrease in park/playground utilization among WIC participants in LAC in 2020 aligns with the results of data collected from 35 states which found that more than 50 % of parents of children 5 to 8 years of age reported that their child engaged in somewhat or much less physical activity early in the COVID-19 pandemic relative to immediately before the pandemic (Dunton et al., 2020). The decline in park/playground utilization frequency in this study was present among children of all race/ethnicity groups, in alignment with an international review of the literature on changes in child physical activity during the COVID-19 pandemic in 21 studies, with significant decreases in physical activity identified in 13 studies and non-significant decreases in another 6 (Yomoda and Kurita, 2021). Studies conducted among urban-dwelling adults in North Carolina (Larson et al., 2021) and adolescents across the United States (Jackson et al., 2021) identified significant decreases in park utilization and outdoor recreation during the COVID-19 pandemic, similar to the lower park/playground utilization identified among children<5 years of age in the present study.

Despite the tremendous decrease in odds of more frequent park/playground utilization identified in this study, nearly 30 % of WIC-participating children continued to go to a park/playground every day. This aligns with a prior study that found individuals who consistently used parks before the COVID-19 pandemic were more likely to increase park use during the pandemic (Larson et al., 2021). A similar pattern was identified among all racial/ethnic groups in this study, with a higher proportion of children going to the park every day in 2020 compared to the proportion of every day park/playground users in the combined surveys from 2008 to 2017. This indicates that, even though the proportion of every day park/playground utilization declined from 2017 to 2020 there was a subset of WIC-participating families for whom the COVID-19 pandemic did not decrease park/playground utilization in a way the study was able to assess.

Our study identified racial/ethnic disparities in the frequency of park/playground utilization. Among children 24–59 months of age before 2020, Asian children were 69 % less likely to use parks/playgrounds every day; Black and Spanish-speaking Hispanic children were 86 % and 90 % more likely to use parks/playgrounds every day than English-speaking Hispanic children. A similar disparity was reported in a 2012 study that found Asian children were 49 % less likely to engage in outside play daily than White children (Tandon et al., 2012). That study reported that Black and Hispanic children were less likely to engage in outdoor play than White children (Tandon et al., 2012), conflicting with the present results that before 2020 Black and Spanish-speaking Hispanic children used parks/playgrounds more frequently than English-speaking Hispanic children, and of no disparity between White and English-speaking Hispanic children. Park/playground utilization frequency declined among Asian, Black and Spanish-speaking Hispanic children relative to English-speaking Hispanic children in 2020. Reasons for these differences may relate to household socio-economic characteristics including family income, the type of residence, family size, or to differences in proximity of park/playground facilities to the residence by race/ethnicity (Yomoda and Kurita, 2021). Declines in physical activity during the COVID-19 pandemic were more pronounced among children of foreign nationals in Spain (Medrano et al., 2021), and a similar pattern in the United States may have contributed to decreased frequency of park/playground utilization among Spanish-speaking Hispanic relative to English-speaking Hispanic children during 2020.

The large decrease in park/playground utilization identified in 2020 aligns with warnings issued by PA-policy experts early in the COVID-19 pandemic that park/playground restrictions may have an outsized immediate impact on PA, and subsequently on population physical and mental health (Slater et al., 2020). Most state governors in the United States, including the governor of California, issued stay-at-home orders in March 2020 that severely curtailed the mobility of individuals within their jurisdiction (Gostin and Wiley, 2020). It was not until June of 2021 that the stay-at-home order was officially terminated in California, eliminating state restrictions including physical distancing and county-based restrictions related to COVID-19 transmission intensity tiers (State of California- Health and Human Services Agency, 2021). The restrictions enacted early in the COVID-19 pandemic in California, which remained in force with intermittent modification throughout 2020, may have contributed to decreased utilization of parks/playground by restricting access directly, (Los Angeles County Department of Public Health, 2020, Los Angeles County Department of Parks and Recreation, 2020) or by increasing the perceived health risks of park/playground use by individuals who might have otherwise used these public spaces (Curtis et al., 2022).

This study has many noteworthy strengths. It was conducted in large, randomly-selected samples of WIC-participating families in LAC, making the study population representative of the WIC service population in LAC in each survey year. The results were adjusted for household socio-demographic characteristics and contextual indicators for the census tract of residence, which should control for secular trends in neighborhood and WIC-participant characteristics over the study period. The study was limited by the cross-sectional nature of the data, preventing a direct assessment of the impact of COVID-19-related park/playground restrictions on individual utilization of these facilities. Causal inference is precluded by the observational study design. Residual confounding by unknown or unmeasured household or neighborhood characteristics may contribute to the reported associations. The study population is entirely WIC-participating children in LAC, making the population low-income and predominantly Hispanic across all survey years, potentially limiting generalizability to populations that are higher income and lower proportion Hispanic. Finally, surveys were collected from April to August in 2008–2017, while surveys in 2020 were collected from July to December. Seasonal variation in park/playground utilization in the comparison of 2020 to prior survey years cannot be conclusively ruled out, though mild temperatures year-round should minimize seasonality of park/playground usage, and a sensitivity analysis identified no seasonality in the 2020 data.

## 5 Conclusions

Marked decreases in park/playground utilization frequency among children ages 4–23 months and 24–59 months living in low-income, WIC-participating households in LAC could have a tremendous health impact for the children affected. These data suggest that public-health policies enacted early in the COVID-19 pandemic may have contributed to substantial negative externalities, and that future public health restrictions should be informed by targeted efforts in high-risk settings, rather than blanket restrictions across low-risk populations (e.g. young children) and settings (e.g. parks/playgrounds). Given the well-established relationships between play, physical activity and healthy development, the identified park/playground utilization deficit in 2020 could have substantial impacts on the prevalence of overweight or obesity and developmental delays. Future research will be needed to assess whether park/playground utilization increases for children in this age range following the COVID-19 pandemic, or whether these children remain on a low utilization trajectory throughout their childhood. Developmental and weight outcomes associated with these park/playground utilization deficits also need to be evaluated relative to children before the pandemic, and to children born following the conclusion of the pandemic.

## CRediT authorship contribution statement

**Christopher E. Anderson:** Conceptualization, Methodology, Data curation, Software, Formal analysis, Writing – original draft. **Shannon E. Whaley:** Conceptualization, Methodology, Resources, Funding acquisition, Writing – review & editing.

## Declaration of Competing Interest

The authors declare that they have no known competing financial interests or personal relationships that could have appeared to influence the work reported in this paper.

## Data Availability

The data that has been used is confidential.
